# Design and performance of a new VIS–VUV photoluminescence beamline at UVSOR-III

**DOI:** 10.1107/S1600577513032931

**Published:** 2014-01-28

**Authors:** Kazutoshi Fukui, Ryu-ichi Ikematsu, Yoshinori Imoto, Mamoru Kitaura, Kazumichi Nakagawa, Takao Ejima, Eiken Nakamura, Masahiro Sakai, Masami Hasumoto, Shin-ichi Kimura

**Affiliations:** a Faculty of Engineering, University of Fukui, 3-9-1 Bunkyo, Fukui 910-8507, Japan; b Faculty of Science, Yamagata University, 1-4-12 Kojirakawamachi, Yamagata 990-8560, Japan; c Graduate School of Human Development and Environment, Kobe University, 3-11 Tsurukabuto, Nada-ku, Kobe, Hyogo 657-8501, Japan; d Institute of Multidisciplinary Research for Advanced Materials, Tohoku University, 2-1-1 Katahira, Aoba-ku, Sendai, Miyagi 980-8577, Japan; e UVSOR Facility, Institute for Molecular Science, 38 Nishigo-Naka, Myodaiji, Okazaki, Aichi 444-8585, Japan

**Keywords:** beamline, normal-incidence monochromator, photoluminescence

## Abstract

A new bending-magnet beamline with a 2.5 m normal-incidence monochromator has been constructed to serve with a light source in the visible–vacuum-ultraviolet region for photoluminescence, transmission and reflection spectroscopies of solids at the UVSOR-III 750 MeV synchrotron radiation light source. The aim is to pave the way to establishing a beamline with high photon flux, high brilliance, high energy-resolution, high linear-polarization and low higher-order light. To obtain high photon flux and brilliance, the acceptance angle of the bending-magnet radiation was designed to be 40 mrad (H) × 14 mrad (V) and the post-mirror system employed Kirkpatrick–Baez optics. The incidence angle of the incoming light to the optical elements, except to the gratings, was set to a grazing angle in order to keep a degree of linear polarization. For achieving high energy-resolution, an off-plane Eagle-type monochromator was adopted. Higher-order unwanted light in the energy range below ∼11 eV was suppressed to be less than 0.1%.

## Abstract 2

A new bending-magnet beamline with a 2.5 m normal-incidence monochromator mainly dedicated to photoluminescence measurements of solids has been constructed at the UVSOR-III.

## 1. Introduction   

Solid-state optical devices in the ultraviolet (UV) region have brought about significant improvements in telecommunication, illumination, environmental and medical applications, and are expected to lead to further progress. For technological and scientific research on such industrially important UV materials, optical spectroscopic instruments that cover a wide photon energy region, from the visible (VIS) to the vacuum-ultraviolet (VUV) regions, are required.

We have developed a new bending-magnet beamline, HOTRLU (High-resOlution Transmission, Reflection and LUminescence spectroscopies of solids in the VIS–VUV region), equipped with a 2.5 m normal-incidence monochromator (NIM) at beamline BL3B of UVSOR-III in order to realise accurate photoluminescence (PL) spectroscopy of such materials in the VIS–VUV regions. HOTRLU has also been constructed as a succession beamline of BL1B (1 m Seya–Namioka-type monochromator beamline), which was closed down at the end of March 2011. HOTRLU is featured as the third specific VIS–VUV beamline at UVSOR-III: a multipurpose beamline (3 m McPherson-type monochromator beamline, BL7B; see Fukui *et al.*, 2001 ▶), a variably polarized angle-resolved photoemission beamline with a 10 m modified Wadsworth-type monochromator (SAMRAI, BL7A; see Kimura *et al.*, 2010 ▶), and a new PL-dedicated beamline (HOTRLU). In this report we describe the design details and present performance of the HOTRLU beamline.

## 2. Design   

The beamline monochromator for PL measurements is required to satisfy a wide photon energy range, giving a high brilliance on the sample, while maintaining a high degree of linear polarization, a high energy-resolution and high purity (inclusion of less higher-order light). Thus we adopted the following target specifications for the beamline using examples from the performance of the BL7B beamline: a photon energy coverage range of 2–24 eV, photon flux (*E*/Δ*E* = 1000) higher than 10^10^ photons s^−1^, energy resolution (*E*/Δ*E*) higher than 10000, beam spot size (H × V) less than 0.8 mm × 0.8 mm, and degree of linear polarization higher than 0.7.

Since a NIM can maintain both incident beam profile and polarization of the outgoing beam, and the reflectivity is functional up to ∼30 eV, it is a good solution for achieving the above beamline specifications. Considering the high-resolution specification, we adopt an off-plane Eagle-type NIM (see Namioka, 1959 ▶). We use a vertical-dispersion grating configuration where both entrance and exit slits are located in the same plane. Even though the reflectivity of the normal incidence is functional up to ∼30 eV, the square of the reflectivity on the higher energy side becomes low and is not appropriate for PL measurements. Therefore, the layout of this beamline needs to consist of a normal-incidence grating with the other optical elements at grazing incidence. This configuration also preserves the degree of linear polarization of the bending-magnet radiation. However, the floor shape at the beamline usually spreads out toward the end of the beamline. This implies that the layout of the NIM beamline with normal-incidence grating configuration is dis­advantageous because user experiment space becomes insufficient. To avoid this, we rotate the monochromator and the post-mirror system in the horizontal plane, and introduce a plane mirror into the pre-mirror system. This mirror also acts as a higher-order cut filter if it is vertically split-coated. The optical layout in the vertical direction is also designed to satisfy the following two conditions: (i) the output beam of the HOTRLU beamline is horizontal, and (ii) the height of the output beam is ∼1200 mm. These conditions facilitate PL experiments on condensed matter. For the PL measurements, not only a high photon flux but also high brilliance is required. Thus, for good focusing at the experimental point we employ Kirkpatrick–Baez optics (see Kirkpatrick & Baez, 1948 ▶). An optical layout simulation was carried out using the ray-tracing program *SHADOW* (see Lai & Cerrina, 1986 ▶).

**Figure 1:**
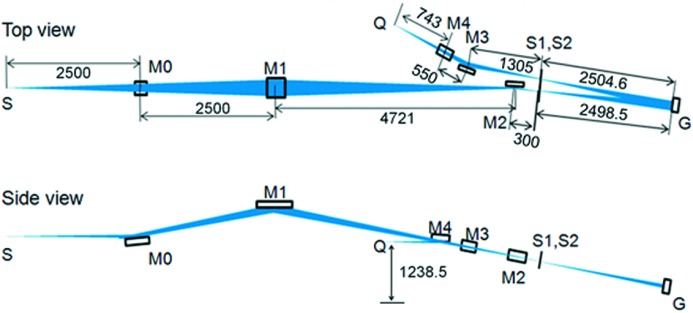
Schematic layout of the HOTRLU. (*a*) Top view and (*b*) side view. Dimensions are given in millimeters.

The adopted parameters of the optical components are listed in Table 1 ▶, and the top and side views are schematically shown in Figs. 1(*a*) and 1(*b*) ▶, respectively. The acceptance angle of the beamline is 40 mrad (H) × 14 mrad (V), which satisfies the prerequisite photon flux. M0 is a plane mirror for changing the direction of the bending-magnet radiation at the point S and for reducing heat load. A radiation shield wall is located in between the M0 and M1 mirrors, where a toroidal mirror M1 focuses incoming bending-magnet radiation to the entrance slit S1 (1:1) to prevent the focal spot shape from deforming owing to a wide acceptance angle. A plane mirror M2 reflects the light in the horizontal direction to reserve working space around the sample (Q) and to correct horizontal beam misalignment, as mentioned above. An off-plane Eagle-type monochromator with focal length of 2.5 m consists of S1, gratings G and exit slit S2. The monochromator satisfies both *F*-number matching to the pre-mirror system as well as the prerequisite resolution specification. We use three gratings (G1, G2 and G3) with different grooves, blazing angles and coatings to cover the wide photon energy region from 2 to 30 eV. The horizontal deviation angle of G is 4°. The total rotation angle (angle resolution) of the gratings and total linear travel (travel resolution) of the grating chamber were 6° (0.036°) and 20 mm (0.01 mm), respectively. Four optical low-energy-pass filters (LPFs), LiF, quartz, WG32 and OG52, are available and a motorized linear actuator can set one of them just after S2, to reject higher-order light in the low-energy region. The post-mirror system with Kirkpatrick–Baez optics consists of two spherical mirrors M3 and M4 which condense incoming light from S2 horizontally and vertically. The M4 mirror also realigns the beam parallel to the floor. Finally, the horizontally propagating beam is focused onto the Q point. Both the VIS–UV and VUV spectrometers with CCD sensors for PL measurements can be placed in the sample chamber. The calculated degree of linear polarization on the paraxial ray of this layout is expected to be higher than 0.7 over the whole energy region.

## 3. Present performances   

**Figure 2:**
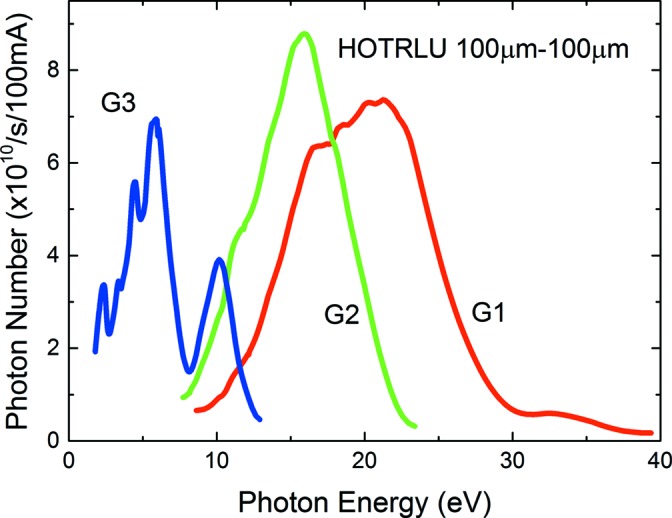
Throughput spectra of the HOTRLU beamline.

**Figure 3:**
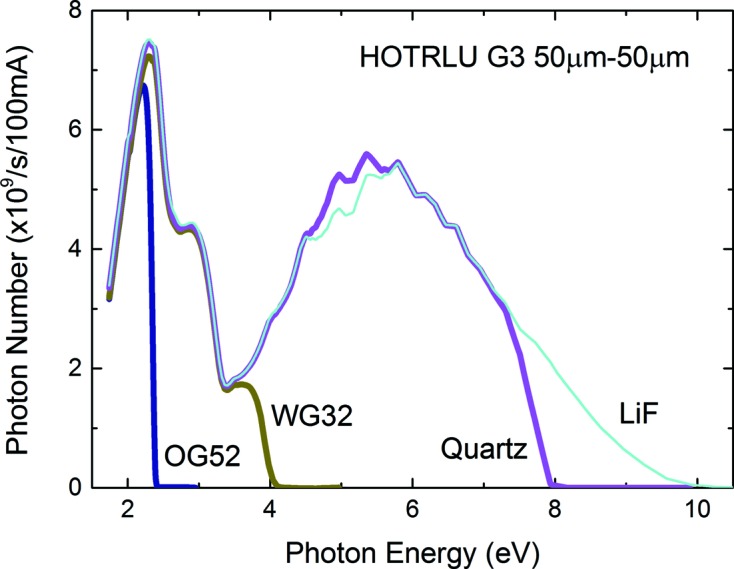
Throughput spectra with the G3 grating described in Table 1 ▶, measured with a low-energy-pass filter.

Fig. 2 ▶ shows the throughput spectra measured at the Q point by a Si photodiode (SPD; AX-UV100, IRD). The slit widths of S1 and S2 were set to 100 µm. The photon number estimated using the typical quantum efficiency of AX-UV100 was ∼1 × 10^10^ photons s^−1^ or more over the whole photon energy rage from 1.7 eV to ∼35 eV, at a beam current of 300 mA, which is the typical current of the top-up operation of UVSOR-III. Fig. 3 ▶ shows the system throughput with the G3 grating for the above-mentioned LPF variation. The LPF cut-off photon energies are 11.8 eV (LiF), 7.7 eV (quartz), 3.9 eV (WG32) and 2.4 eV (OG52). We have confirmed that the intensity of the higher-order light is less than 0.1% of that of the first-order light; thus we can obtain high-purity monochromated light by using the LPFs in the G3 range below ∼11 eV. This photon energy range is very important for the PL measurement of solids.

**Figure 4:**
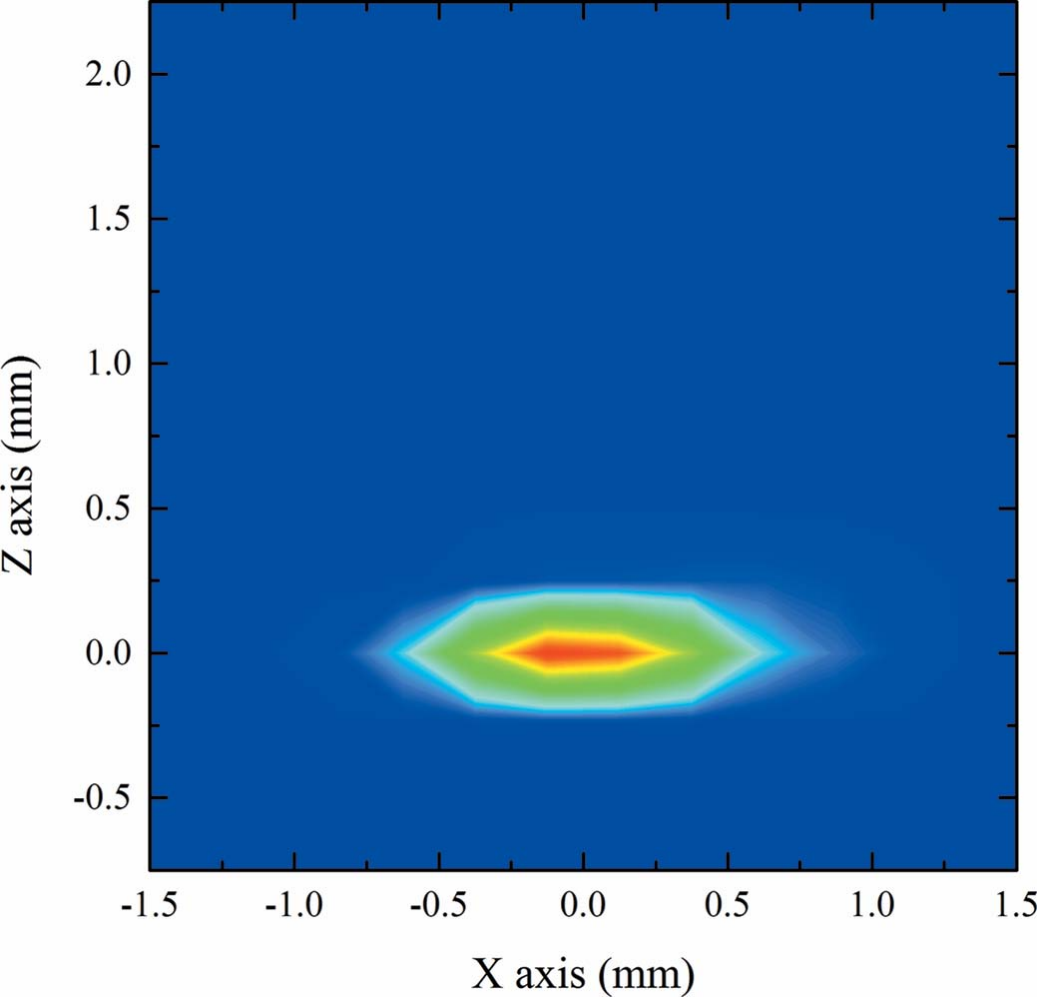
Beam profile contour map image at the sample position measured at 100 nm using the G2 grating described in Table 1 ▶.

Fig. 4 ▶ shows a contour map representing the beam spot profile at the Q point, obtained by raster scan of a metallic plate with a 0.2 mm-diameter pinhole placed just before the SPD and measuring output current. The profile shown in Fig. 4 ▶ was recorded using G2 at ∼12 eV (∼100 nm). The full width at half-maximum (FWHM) of the profile was evaluated to be 0.75 mm (H) × 0.25 mm (V). Beam profiles for the other gratings were similar to that of G2 shown in Fig. 4 ▶. However, for all gratings, profile deformations were clearly observed at the low photon energy limit.

**Figure 5:**
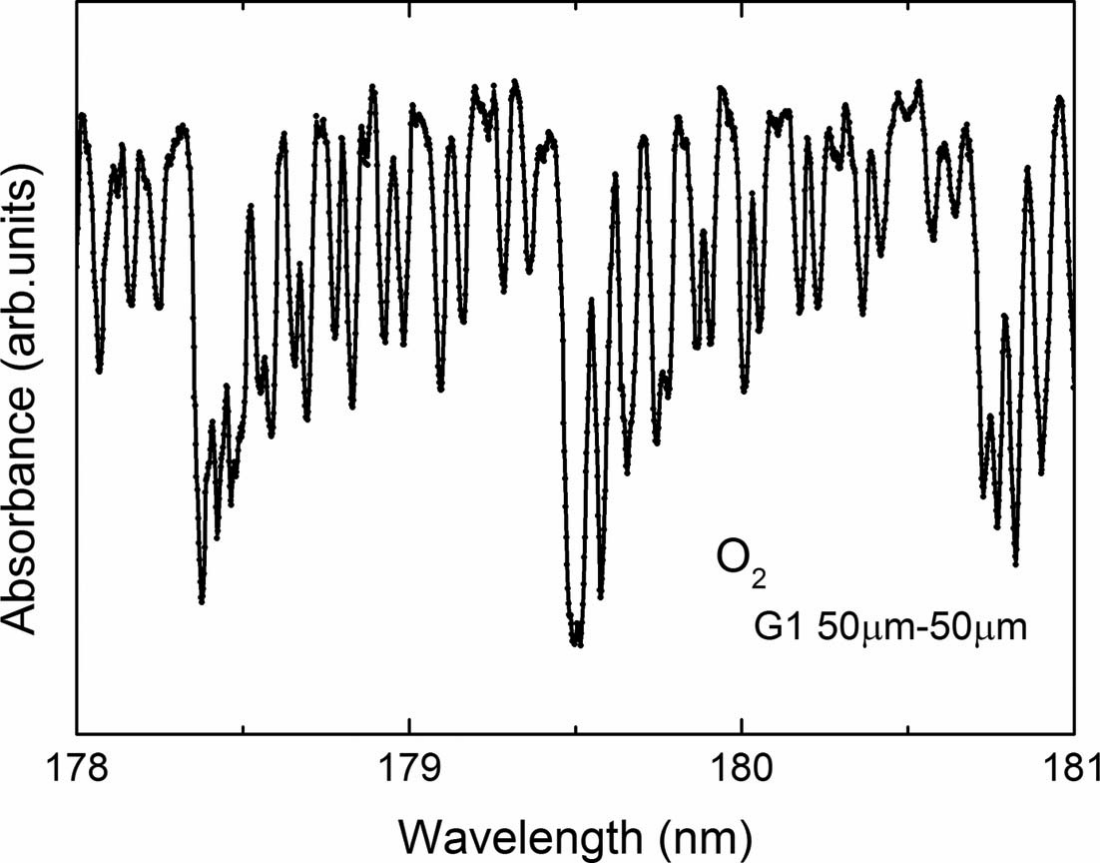
Absorption spectrum in the spectral range of the Schumann–Runge oxygen molecule band.

The absorption spectrum of oxygen molecules in the spectral range of the Schumann–Runge bands, measured with G1 at slit widths S1 and S2 of 50 µm, is shown in Fig. 5 ▶. Here the horizontal axis, representing wavelength, is not calibrated. In this spectrum the energy (wavelength) resolution (*E*/Δ*E*) for 50 µm slit opening is roughly 5000 or more.

**Figure 6:**
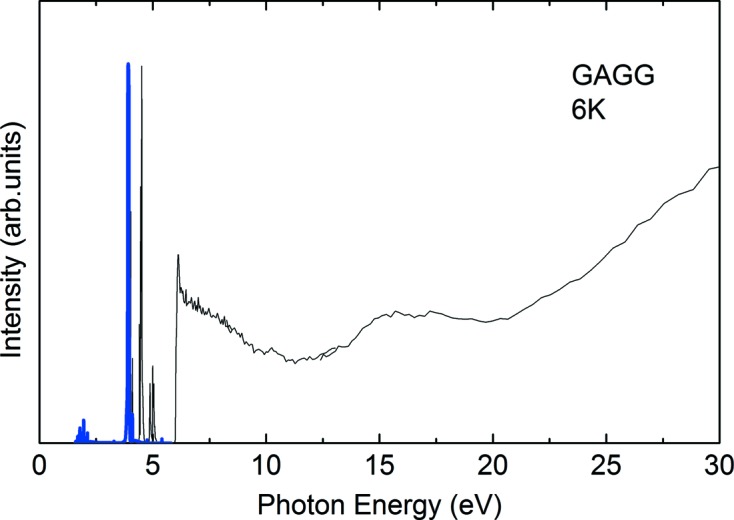
Photoluminescence (thick blue line) and photoluminescence excitation (thin black line) spectra of a Gd_3_Ga_3_Al_2_O_12_ (GAGG) single crystal measured at 6 K. The excitation energy of the PL spectrum and the emission energy of the PLE spectrum are 6.52 eV and 3.92 eV, respectively.

In Fig. 6 ▶ we show typical PL and PL excitation (PLE) spectra of a Gd_3_Ga_3_Al_2_O_12_ crystal (GAGG; see Kamada *et al.*, 2012 ▶) taken at the HOTRLU beamline. The sample size and the measurement temperature were 10 mm × 5 mm and 6 K, respectively. The PL spectrum (thick blue line) was measured using a 30 cm Czerny–Turner monochromator with an entrance slit width of 100 µm and with a liquid-nitrogen-cooled back-illuminated CCD. The excitation energy was set to 6.52 eV by G3, and both S1 and S2 were set to 300 µm. A sharp PL peak at 3.92 eV was assigned to the 4*f* intra-atomic emission of Gd^3+^ ions. The thin black line is a PLE spectrum of the sharp emission peak at 3.92 eV, which was measured up to 30 eV by successively replacing the grating among the G3, G2 and G1 gratings. In the PLE spectrum, sharp peaks owing to 4*f* intra-atomic absorptions of Gd^3+^ ions and a clear sharp edge corresponding to the band-to-band absorption of the GAGG are clearly observed at 4–5 eV and at 5.96 eV, respectively. A gradual increase of the intensity in the higher energy range (>20 eV) is also observed, which is interpreted as the appearance of multiple electron–hole pairs in GAGG.

## 4. Summary   

A new bending-magnet beamline at BL3B has been constructed (HOTRLU) equipped with a 2.5 m normal-incidence monochromator. The present performance of HOTRLU is as follows (the parameters in square brackets are our target performance at the design stage): photon energy coverage, 1.7–31 eV [2–24 eV]; photon flux (*E*/Δ*E* = 1000), ≥10^10^ photons s^−1^ (300 mA)^−1^ over a whole region [≥10^10^ photons s^−1^ (300 mA)^−1^]; energy resolution (*E*/Δ*E*) (G1, S1 50 µm, S2 50 µm), ≥5000 [≥10000]; beam spot size at a sample position (H × V), ∼0.75 mm × 0.25 mm [≤0.8 mm × 0.8 mm]. Both the polarization evaluation and the resolution improvement are future issues.

## Figures and Tables

**Table:** 

Pre-mirrors	Incidence angle ()	Radius (mm)	Dimensions (mm)	Coating material
M0	85	(plane)	110 300	Au
M1	81.1	773.60 32300 (toroidal)	210 280	Au
M2	87	(plane)	30 440	Au

**Table:** 

Gratings	Deviation angle ()	Radius (mm)	Dimensions (mm)	Coating material	Grooves (mm^1^)
G1	4	2500	40 110	Au	1200
G2	4	2500	40 110	Pt	600
G3	4	2500	40 110	Al	300

**Table:** 

Post-mirrors	Incidence angle ()	Radius (mm)	Dimensions (mm)	Coating material
M3	82	9390 (spherical)	40 400	Au
M4	86.5	17090 (spherical)	50 270	Au
